# Caffeic Acid and Metformin Inhibit Invasive Phenotype Induced by TGF-β1 in C-4I and HTB-35/SiHa Human Cervical Squamous Carcinoma Cells by Acting on Different Molecular Targets

**DOI:** 10.3390/ijms19010266

**Published:** 2018-01-16

**Authors:** Malgorzata Tyszka-Czochara, Malgorzata Lasota, Marcin Majka

**Affiliations:** 1 Department of Food Chemistry and Nutrition, Faculty of Pharmacy, Jagiellonian University Medical College, Medyczna 9, 30-688 Krakow, Poland; 2 Chair of Medical Biochemistry, Faculty of Medicine, Jagiellonian University-Medical College, Kopernika 7, 31-034 Krakow, Poland; malgorzata.lasota@uj.edu.pl; 3 Department of Transplantation, Faculty of Medicine, Jagiellonian University Medical College, Wielicka 258, 30-688 Krakow, Poland; mmajka@cm-uj.krakow.pl

**Keywords:** caffeic acid, metformin, epithelial-mesenchymal transition, cervical cancer, epithelial markers, mesenchymal markers, metastasis, hypoxia, carbonic anhydrase IX

## Abstract

During the progression of epithelial cancer, the cells may lose epithelial markers and gain mesenchymal phenotype via Epithelial-Mesenchymal Transition (EMT). Such transformation of epithelial cancer cells to mesenchymal-like characteristic benefits plasticity and supports their ability to migrate. The aim of this study was to evaluate the influence of natural compound Caffeic Acid (CA) alone and in combination with antidiabetic drug Metformin (Met) on metastatic progression of two human cervical squamous cell cancer lines, C-4I and HTB-35/SiHa cells. EMT program was triggered by exposition of both epithelial cell lines to TGF-β1. Gene expression patterns related to epithelial/mesenchymal phenotype were evaluated by Real-Time PCR analysis and the protein amount was detected by western blot. The treatment of human squamous cancer cells with CA and with Met, suppressed the motility of cells and the effect depended on a particular cell line. Both compounds regulated the EMT process in C4-I and HTB-35 cells by interfering with different molecular targets. In TGF-β1-stimulated C4-I cells, CA suppressed the expression of mesenchymal transcription factor SNAI1 which resulted in enhanced expression of epithelial markers E-cadherin, Occludin and Claudin. Additionally, CA blocked *MMP-9* and upregulated *TIMP-*1 expression, a specific inhibitor of MMP-9. In HTB-35 cells stimulated with TGF-β1, Met decreased the expression of Vimentin. By suppressing hypoxia master regulator *HIF-1α*, Met caused downregulation of *CAIX*, an enzyme involved in metastasis of aggressive malignant cells. In this study we showed that CA and Met inhibited EMT process in cancer cells via different mechanisms. However, when applied together, compounds exerted the greater effect on EMT than each compound alone. This is the first report revealing that CA alone and co-treated with Met may reverse mesenchymal phenotype of TGF-β1-treated cervical tumor cells and we believe that the use of the two small molecules may be considered as a potential therapeutic approach for metastatic cervical cancer.

## 1. Introduction

Squamous cell carcinoma is the most common cervical cancer in women and accounts for almost 80% of cervical carcinomas in this population [1]. Along with the advent of human papilloma virus (HPV) vaccines, the primary prevention of cervical malignancy has become more successful; however, survival and prognosis are poor, particularly due to cancer metastasis [2]. Considering the short survival period in patients with recurrent or metastatic cancer, there is an urgent need for improvement of existing therapies for cervical malignancy [3].

Malignant cell transformation consists of a series of processes resulting in the ability of cells to migrate and invade other tissues. When polarized epithelial cells gain invasive characteristic, they lose epithelial markers, especially cell-cell adhesion molecules, such as E-cadherin, Occludin, Claudin and β-catenin, and acquire expression of mesenchymal markers, such as Vimentin, which finally results in the activation of Epithelial-Mesenchymal Transition (EMT) program [4]. The conversion of cancer cells function and morphology to mesenchymal-like phenotype benefits the plasticity and facilitates leaving of the primary site and disseminating to the secondary sites through blood or lymph vessels.

Emerging data suggest that targeting EMT with small molecules derived from plants, such as Caffeic Acid (3,4-dihydroxycinnamic acid, CA), may be an effective chemopreventive approach in various cancers. CA is one of the major hydroxycinnamic acids in the human diet. It occurs in various foods, including herbs and beverages such as coffee and red wine. In the human body, CA may be produced by the hydrolyzation of chlorogenic acid, a phenolic acid abundant in plants. The anticancer activity of CA and its derivatives was demonstrated in vivo [5] as well in vitro [6,7], also against gynecological carcinomas [8]. CA and its derivatives may inhibit the migratory capacity of cancer cells via several mechanisms, including inhibition/activation of transcription factors such as nuclear factor κ-light-chain-enhancer of activated B cells (NFκB) and regulation of Akt/mTOR signaling pathway [9,10]. It was reported that CA reversed TGF-β1- induced EMT in human tumor cell lines [11] and alleviated prostate malignant cells aggressiveness via non-canonical Wnt signaling [5]. CA and its derivatives suppressed EMT process via inhibition of Vimentin and upregulation of E-cadherin, as has been shown in pancreatic [3] and malignant keratinocyte cells [7]. However, the effect of this natural compound on the migratory capacity of neoplastic cervical cancer cells is still unknown. Metformin (dimethylbiguanide, Met) used in humans for the treatment of type 2 diabetes, was shown to restrain EMT in highly metastatic cervical HTB-35/SiHa cells [3] Met, similarly to CA, may prevent TGF-β1- induced EMT and cell invasion by controlling of mesenchymal/epithelial markers expression and by regulation of intracellular signaling pathways triggering cell death [12,13]. The experimental approach used in this study is based on the hypothesis that transforming growth factor β1 (TGF-β1) causes a phenotypical transformation of epithelial cervical cancer cells into mesenchymal-like cells, with subsequent increase in motility of the cells. The two human cervical squamous cell cancer lines, C-4I and HTB-35/SiHa, were selected to investigate the in vitro effects of CA and Met. Both cell lines expressed epithelial characteristic, but expression of E-cadherin and Vimentin differed depending on a particular cell line. In this study we aimed to find out, if exposition of cancer cells to CA and Met may suppress TGF-β1-induced EMT phenotype of cancer cells. We tried to evaluate whether both compounds act on EMT process in C-4I and HTB-35 cells via the same or different regulatory proteins and, based on these findings, assess if simultaneous treatment of cells with CA and Met may exert stronger effect than a single drug. A wound healing assay was also conducted to analyze the influence of CA and Met on motility of the cells. In hypoxic conditions, we tested whether drugs may suppress HIF-1α and regulate HIF-1α downstream protein CAIX.

## 2. Results

### 2.1. Transforming Growth Factor Beta 1 (TGF-β1) Induces Epithelial-to-Mesenchymal Transition (EMT) in C4-I and HTB-35 Cells

**Figure 1:**
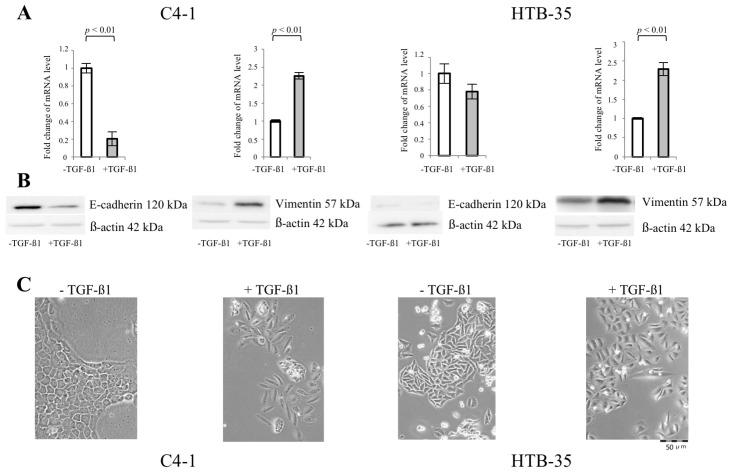
TGF-β1 induces Epithelial-to-Mesenchymal Transition (EMT) in C4-I and HTB-35 cells (**A**–**C**). The human cervical squamous cell cancer lines, C-4I and HTB-35 cells were incubated for 48 h with the addition of 10 ng/mL of TGF-β1. The untreated cells were grown in the same conditions and used as controls. Real-time PCR analysis revealed significant decrease in E-cadherin transcript level relative to untreated control at *p* < 0.01 in TGF-β1-stimulated C-4I cells, while in HTB-35 the drop in mRNA level for E-cadherin was not statistically significant at *p* < 0.05. Note that TGF-β1 caused significant increase in the expression of Vimentin in both cancer cell lines, as measured using qPCR ((**A**), *p* < 0.01 vs. untreated control for C-4I cells and *p* < 0.01 vs. untreated control for HTB-35 cells) and demonstrated with Western blot analysis ((**B**), 20 μg of total cell lysates were subjected to SDS-PAGE followed by immunoblotting and chemiluminescent detection; β-actin was used as loading control). The experiments were repeated three times with similar results; the Real-Time PCR data were presented as mean values ± SD (**A**), a representative immunoblots were shown (**B**). The incubation of the cells with TGF-β1 for 48 h caused morphological changes in both cell lines, as shown in phase contrast microphotographs (**C**). The enhanced scattering of C-4I and HTB-35 cells was observed following TGF-β1 treatment.

EMT is characterized by a drop in the expression of proteins involved in junctional complexes related to polarized epithelial phenotype, such as E-cadherin, with concomitant upregulation of mesenchymal proteins, especially reorganizing cytoskeleton, such as Vimentin. We exposed cervical cancer squamous cell lines C4-I and HTB-35 to 10 ng/mL of TGF-β1 for 48 h. Unstimulated cells cultured in the same conditions were appropriate controls. Western blot and qPCR analyses were performed to determine the expression of EMT-specific proteins in C4-I and HTB-35 cells (Figure 1A,B). In unstimulated C4-I cells, strong E-cadherin (*CDH1*) expression was detected, whereas Vimentin (*VIM*) was barely expressed. The incubation of C4-I cells with TGF-β1 caused downregulation of E-cadherin (*p* < 0.1 vs. untreated cells) and enhanced expression of Vimentin (*p* < 0.1 vs. untreated cells). As shown in Figure 1C, C4-I cells displayed an epithelial appearance [14]. Following exposure to TGF-β1 for 48 h, the cells started to dissociate from monolayer. The unstimulated HTB-35 cells expressed Vimentin (Figure 1A,B), while in TGF-β1-stimulated HTB-35 cells the expression of Vimentin was further enforced (*p* < 0.1 vs. untreated cells). At the same time, the enhanced scattering in TGF-β1-stimulated HTB-35 cells was observed (Figure 1C). E-cadherin was weakly expressed in HTB-35 cells and the treatment with TGF-β1 caused no distinct alteration of the expression of the protein (Figure 1A,B).

### 2.2. CA Attenuates the Migratory Capacity of C4-I and Met Inhibits Motility of HTB-35 Cells

**Figure 2:**
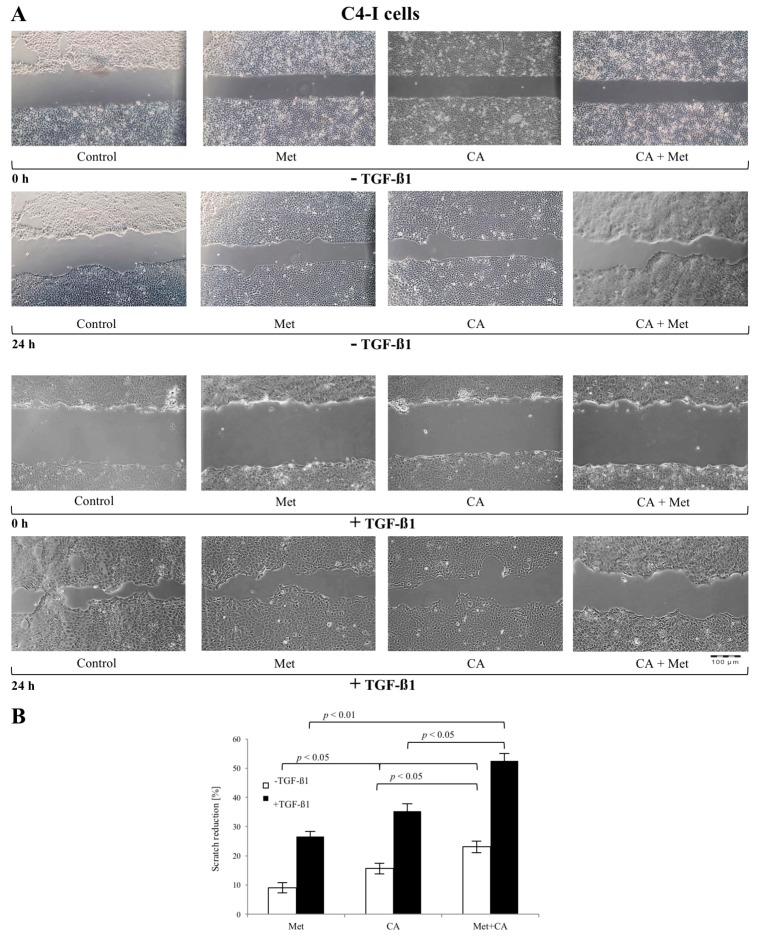
The effect of Caffeic Acid (CA) and Metformin (Met) on migration of C4-I cells in vitro (**A**,**B**). C4-I cells were cultured to sub-confluency and then a scratch was made on the monolayer of cells. Then the cells were incubated with addition of tested compounds (CA at 100 μM and/or Met at 10 mM) and with/without 10 ng/mL TGF-β1 for 24 h. For each scratch the images were obtained by an inverted light microscope (Olympus IX-70, Hamburg, Germany) at 0 h and 24 h. The changes of area of each wound was measured using Image J (v1.44; National Institutes of Health, Bethesda, MD, USA) and the migration rate was quantified as a change of the scratch reduction. Note that CA/Met caused the greatest scratch reduction in TGF-β1-treated cells ((**B**), *p* < 0.01 for CA/Met vs. control, *p* < 0.05 for CA/Met vs. Met, *p* < 0.05 for CA/Met vs. CA) as well as in TGF-β1-untreated cells ((**B**), *p* < 0.05 for CA/Met vs. control, *p* < 0.05 for CA/Met vs. Met, *p* < 0.05 for CA/Met vs. CA). All bars show the mean value of three experiments and error bars represent standard error of the mean (**B**).

**Figure 3:**
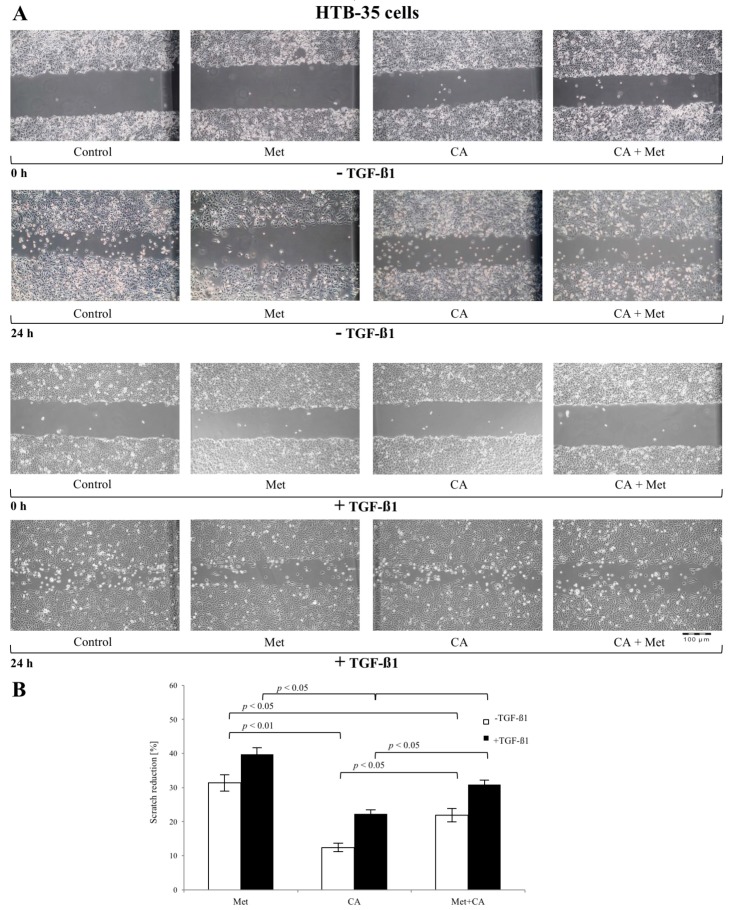
The effect of Caffeic Acid (CA) and Metformin (Met) on HTB-35 cells migration in vitro (**A**,**B**). HTB-35 cells were cultured to sub-confluency and then an injury line was made on the monolayer of cells. Then the cells were incubated with addition of tested compounds (CA at 100 μM and/or Met at 10 mM) and with/without 10 ng/mL TGF-β1 for 24 h. Representative images of the scratch assay conducted on the HTB-35 cell line following treatment of cells with CA at concentration of 100 µM, Met at 10 mM or both compounds were presented. The images were captured at 0 and 24 h after scratching (**A**). The effect of CA and/or Met on cell motility was determined by wound healing assay and presented as reduction of each scratch after 24 h of incubation ((**A**), the Image J program was used to analyze the changes in each wound area; Image J v1.44; National Institutes of Health, Bethesda, MD, USA). Note that Met exerted greater effect than CA ((**B**), *p* < 0.05 for CA vs. Met) and CA/Met in HTB-35 cell line ((**B**), *p* < 0.05 for Met vs. Met/CA). Data shown here were from a representative experiment repeated three times with similar results (**A**,**B**). Quantification of the scratch assay experiments are presented as mean values ± SD (**B**).

Scratch assays were performed to determine the possible influence of CA and Met on the functional effects of EMT in C4-I and HTB-35 human squamous cell cancer lines. The sub-confluent cell cultures were incubated with CA and/or Met for 24 h. In parallel, cultures treated with tested compounds ware exposed to 10 ng/mL of TGF-β1. As shown in Figure 2A and Figure 3A, TGF-β1 augmented migration of both cell lines when compared to unstimulated controls. The 100 µM CA treatment reduced the invasion potential of C4-I cells (Figure 2B, *p* < 0.05 vs. untreated cells) and HTB-35 cells (Figure 3B, *p* < 0.05 vs. untreated cells). The exposition of cells to 10 mM Met also significantly facilitated the closure of the denuded area in C4-I cell line (Figure 2B, *p* < 0.05 vs. untreated cells) and in HTB-35 cell line (Figure 3B, *p* < 0.05 vs. untreated cells). Comparing the effect of tested compounds on scratch reduction in the two cell lines, CA inhibited the healing process in C4-I cells more effectively than Met (Figure 2B, *p* < 0.05 for CA vs. Met) while Met exerted effect greater than CA in HTB-35 cell line (Figure 3B, *p* < 0.05 for CA vs. Met). In C4-I cells treated with TGF-β1 CA/Met caused the greatest scratch reduction (up to 50%). What is more, co-treatment had greater impact on motility of the cells than each compound alone (Figure 2B, *p* < 0.05 for CA/Met vs. CA, *p* < 0.05 for CA/Met vs. Met). In HTB-35 cells Met caused a 40% reduction of cell scratch and the most effective attenuation of cell movement (Figure 3B, *p* < 0.05 for Met vs. CA, *p* < 0.05 for Met vs. CA/Met).

We examined the influence of CA and Met with and without addition of 10 ng/mL of TGF-β1 on the proliferation of C4-I and HTB-35 cells. The confluent cell cultures were exposed to compounds for 24, 48 and 72 h. The assessment of cell number revealed that CA and Met slightly reduced the growth of C4-I and HTB-35 cells after 24 and 48 h of exposure (Figure S1A). Since CA at 100 µM and Met at 10 mM significantly decreased the migratory capability of both cell lines cells, with only minor inhibition of growth (Figure S1), we subjected these concentrations to further investigation.

### 2.3. CA and Met Treatment of C4-I Cells Increases Epithelial Adhesive Markers and Decreases Mesenchymal Transcription Factors Regulating EMT

**Figure 4:**
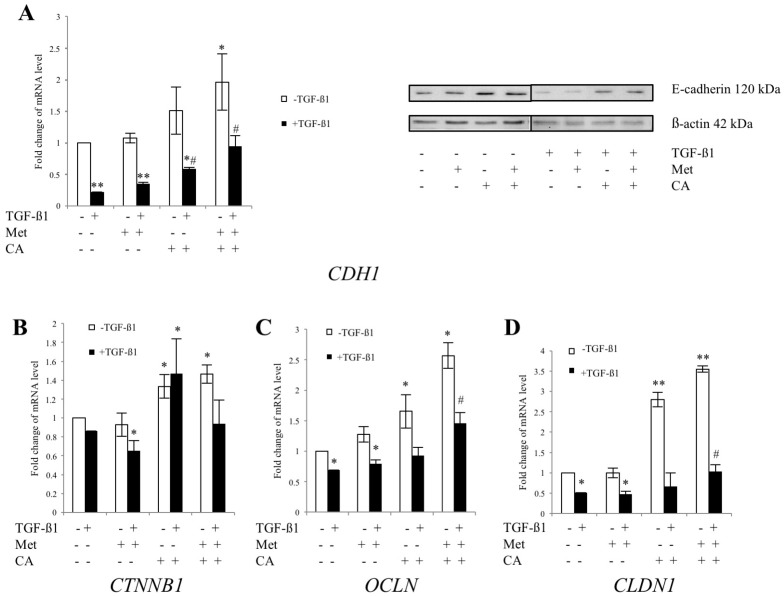
The effect of Caffeic Acid (CA) and Metformin (Met) on expression of epithelial markers in C4-I cells. The cells were treated for 48 h with TGF-β1 (10 ng/mL) plus CA (100 μM) and/or Met (10 mM). In parallel, cultures treated with tested compounds, but without TGF-β1 were prepared. Analysis of mRNA level for E-cadherin (*CDH1*), β-catenin (*CTNNB1*), Occludin (*OCLN*) and Claudin (*CLDN1*) was performed with Real-time PCR ((**A**) left panel, (**B**–**D**), respectively). Protein levels of E-cadherin were analyzed by western blot and shown in the right panel of Figure 4A (20 μg of total cell lysates were subjected to SDS-PAGE followed by immunoblotting and chemiluminescent detection, and β-actin was used as the protein loading control, the details described in Material and Methods). Note that the greatest upregulation for E-cadherin transcript was detected when CA was applied with Met ((**A**), left panel); the same effect was found for Occludin (**C**) and Claudin (**D**) (* *p* < 0.05 vs. control for E-cadherin, * *p* < 0.05 vs. control for Occludin, * *p* < 0.01 vs. control for Claudin). For qPCR the data were normalized against *GAPDH* transcript as a reference gene and levels of RNA expression were determined with the 2^−ΔΔ*C*t^ method (* *p* < 0.05 and ** *p* < 0.01 vs. control, ^#^ *p* < 0.05 vs control with TGF-β1). Experiments were repeated three times with similar results and presented as mean values ± SD.

As the co-treatment with CA and Met the most effectively delayed the motility of C4-I cells, we evaluated whether the compounds may exert changes in expression of adhesion molecules representing an epithelial phenotype, such as E-cadherin, β-catenin, Occludin and Claudin. The effect of CA and Met on changes in mRNA level for the epithelial markers was measured in cultures of C4-I cells incubated for 48 h with the compounds and with addition of 10 ng/mL of TGF-β1. In parallel, for comparative reasons each culture was grown without addition of TGF-β1. CA upregulated E-cadherin, Occludin and Claudin (*p* < 0.05 vs. control) in TGF-β1-stimulated/unstimulated cells. However, as shown in Figure 4, CA/Met treatment caused the greatest increase in the expression of mRNA for *CDH1* (*p* < 0.05 vs. control), *OCLN* (*p* < 0.05 vs. control) and *CLDN1* (*p* < 0.05 vs. control) genes.

**Figure 5:**
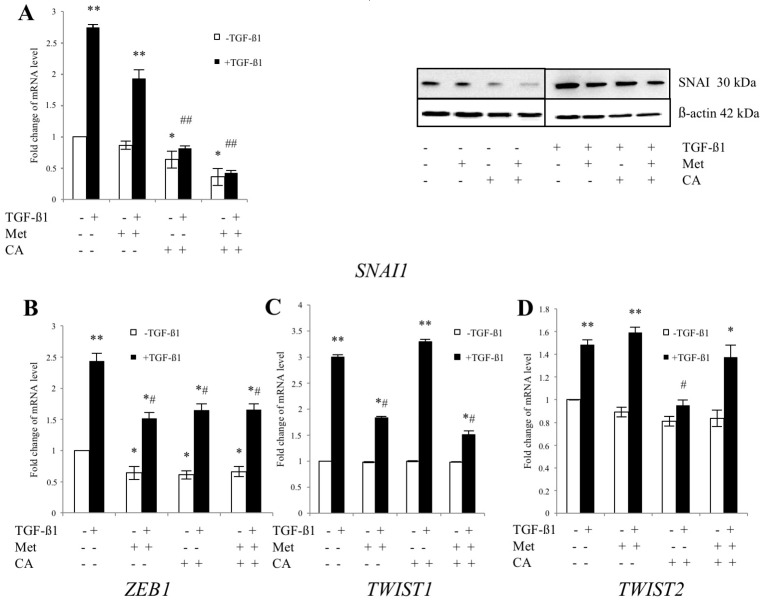
The effect of Caffeic Acid (CA) and Metformin (Met) on expression of transcription factors regulating EMT in C4-I cells. The cells were treated for 48 h with TGF-β1 (10 ng/mL) plus CA (100 μM) and/or Met (10 mM). In parallel, cultures of both cell lines were incubated with tested compounds, but without addition of TGF-β1. Expression of critical EMT promoters *SNAI* (**A**), *ZEB1* (**B**)*; TWIST1* (**C**) and *TWIST2* (**D**) was evaluated at mRNA level by qPCR. For Snail1 protein levels were detected by western blot ((**A**), right panel). Immunoblots were prepared following SDS-PAGE separation of cell lysates as described in Materials and Methods (20 μg of total cell lysates were subjected to SDS-PAGE followed by immunoblotting and chemiluminescent detection, and β-actin was used as the protein loading control). In TGF-β1-stimulated cells the expression ((**A**), left panel) and protein amount ((**A**), right panel) of SNAI was significantly downregulated by CA and CA/Met). Met downregulated *TWIST-1* expression and CA decreased mRNA for *TWIST2.* The data were normalized against *GAPDH* transcript as a reference gene and levels of RNA expression were determined with the 2^−ΔΔ*C*t^ method (* *p* < 0.05 and ** *p* < 0.01 vs. control, ^#^ *p* < 0.05 and ^##^ *p* < 0.01 vs. control with TGF-β1). Bar graph is representative of the relative mean transcript abundance ± SD for three experiments. Results were presented from three independent experiments.

To further elucidate the mechanism of action of compounds, we examined the effect of CA and Met on the expression of mesenchymal transcriptional factors. We found that in TGF-β1-stimulated cells expression of SNAI1, a strong repressor of E-cadherin expression, was downregulated by CA (*p* < 0.05 vs. control) and CA/Met treatment (*p* < 0.01 vs. control). CA/Met inhibited *SNAI1* and upregulated *CDH1* expression after 24 of incubation of C4-I cells in TGF-β1-stimulated/unstimulated cells (Figure S2). As demonstrated in Figure 5, the concomitant action of CA and Met had also the greater impact on *SNAI1* expression following 48 h of exposition of cells to drugs. The expression of *ZEB1* was significantly downregulated by CA (*p* < 0.05 vs. control), Met (*p* < 0.05 vs. control) and CA/Met (*p* < 0.05 vs. control). Met downregulated *TWIST1* expression (*p* < 0.05 vs. control) while CA decreased mRNA for *TWIST2* (*p* < 0.05 vs. control).

### 2.4. CA Downregulate the Expression of MMP-9 and Specific Tissue Inhibitor of Matrix Metalloproteinases TIMP-1 in C4-I Cells

**Figure 6:**
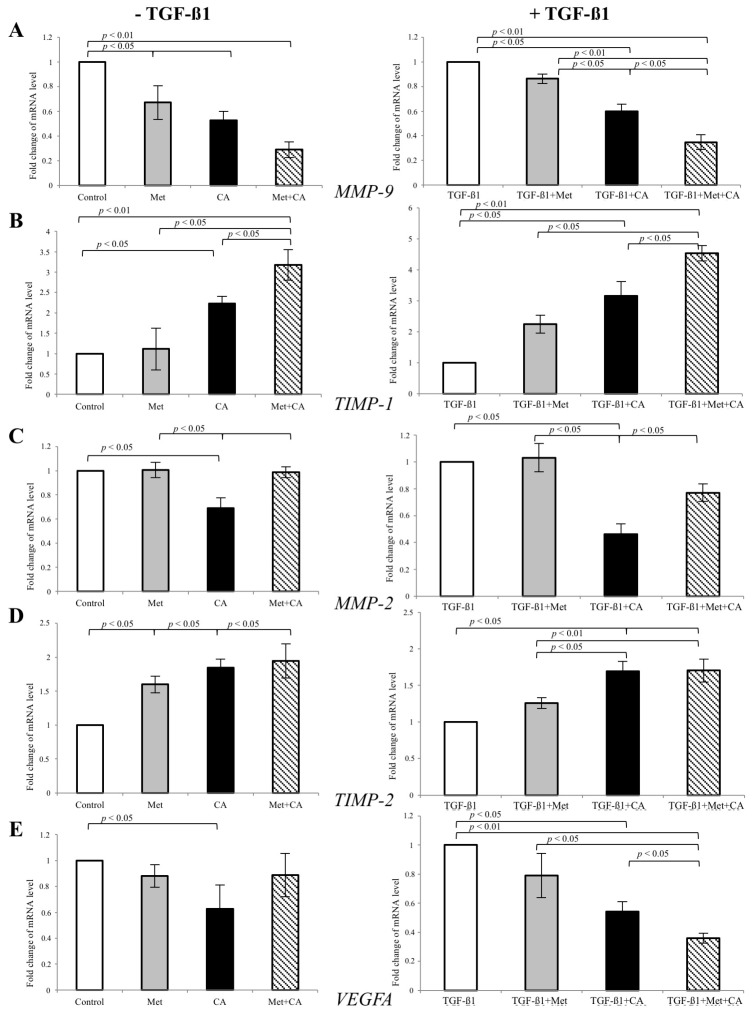
The effect of Caffeic Acid (CA) and Metformin (Met) on the expression of gelatinases *MMP-*9 (**A**) and *MMP-*2 (**C**) and tissue inhibitors *TIMP-1* (**B**) and *TIMP-2* (**D**) in C4-I cells. The cells were incubated with addition of tested compounds (CA at 100 μM and/or Met at 10 mM) and with/without 10 ng/mL TGF-β1 for 48 h. Then mRNA was isolated and purified for Real-time PCR examination. In TGF-β1-stimulated cells, CA and CA/Met caused decrease in mRNA level for *MMP-9* (*p* < 0.01 vs. control) with concomitant increase of mRNA for its specific tissue inhibitor *TIMP-1* (*p* < 0.01 vs. control). The incubation of TGF-β1-treated cells with CA/Met attenuated the expression of angiogenic molecule *VEGFA* (**E**). Data shown here were repeated three times with similar results and presented as mean values ± SD (**B**).

Given that MMP-9 and MMP-2 are critical to cell invasion, we examined the expression of gelatinases at the mRNA level using real-time RT–PCR. C4-I cells were treated with CA and/or Met in the presence of 10 ng/mL of TGF-β1 in medium for 48 h. At the same time, other C4-I cell cultures were exposed only to CA and/or Met, without addition of TGF-β1. The results showed that CA reduced the expression of *MMP-9* (*p* < 0.05 vs. control) in TGF-β1-stimulated cells (Figure 6). As the activity of MMP-9 is regulated by the specific endogenous inhibitor TIMP-1, we determined the effect of tested compounds on mRNA level encoding *TIMP-1*. In fact, the mRNA level for *TIMP-1* was significantly increased in CA treated cells compared to control (*p* < 0.05 vs. control). What is more, CA/Met treatment had greatest inhibitory effect on *MMP-9* expression (*p* < 0.01 vs. control), which was in compliance with the greatest upregulation of *TIMP-1* in cells exposed to both compounds. The qPCR analysis showed that CA suppressed *MMP-2* expression (*p* < 0.05 vs. control) along with upregulation of TIMP-2 (*p* < 0.05 vs. control).

We also determined the effect of CA and Met on the mRNA level for *VEGFA* in TGF-β1-stimulated C4-I cells, since VEGFA is a potent factor facilitating tumor-induced angiogenesis. The mRNA for VEGFA was significantly decreased under exposition of cells to CA (*p* < 0.05 vs. control). Here, CA/Met had the greatest inhibitory impact on *VEGFA* gene expression (Figure 6, *p* < 0.01 for CA/Met vs. control, *p* < 0.05 for CA/Met vs. CA, *p* < 0.05 for CA/Met vs. Met).

### 2.5. Met Attenuates Mesenchymal Marker of Malignant HTB-35 Cells

**Figure 7:**
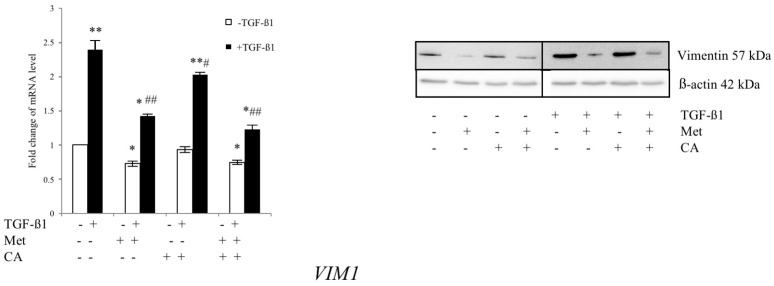
Met suppress the expression of mesenchymal marker Vimentin in HTB-35 cells. The cells were treated with TGF-β1 (10 ng/mL) plus CA (100 μM) and/or Met (10 mM) for 48 h. In parallel, cell cultures were treated with tested compounds but were grown without addition of TGF-β1. The RNA expression was determined with qPCR analysis using the 2^−ΔΔ*C*t^ method (*GAPDH* was a reference gene). The protein levels of Vimentin were detected by western blot (right panel). Immunoblots were prepared following SDS-PAGE separation of cell lysates as described in Materials and Methods (20 μg of total cell lysates were subjected to electrophoresis, β-actin was used as the protein loading control). In TGF-β1-stimulated cells, Met alone and applied with CA significantly decreased the transcript level for Vimentin (*VIM1*). Error bars represent the SD of the mean from triplicate results (qPCR analysis, * *p* < 0.05 and ** *p* < 0.01 vs. control, ^#^ *p* < 0.05 and ^##^ *p* < 0.01 vs. control with TGF-β1).

EMT induction in tumors is associated with increased expression of molecular marker Vimentin. To evaluate the effect of CA and Met on the mRNA level for *VIM1*, HTB-35 cells were exposed to TGF-β1 and tested compounds for 48 h. The appropriate controls with addition of CA and/or Met but without TGF-β1 were made. Following incubation, qPCR and western blot analyses was performed. As presented in 2.1, unstimulated HTB-35 cells exhibited strong expression of Vimentin, while E-cadherin was weakly expressed. Treatment of cells with 10 mM metformin downregulated Vimentin at *p* < 0.01 vs. control in TGF-β1-stimulated cells, as shown in Figure 7. CA caused no distinct alteration of the expression of the mRNA level for *VIM1* (*p* < 0.05 vs. control).

### 2.6. Met Inhibits the Expression of CAIX in HTB-35 Cells under Hypoxic Conditions

**Figure 8:**
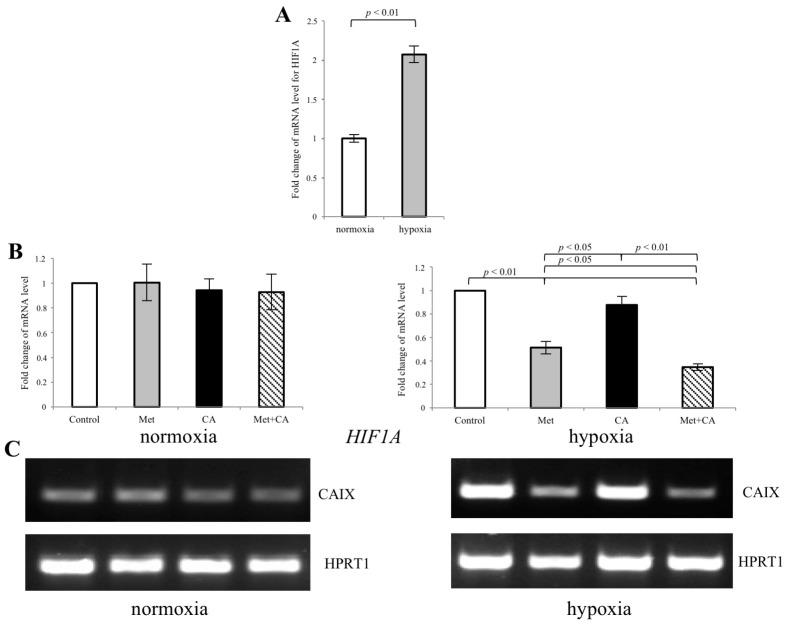
Met inhibits the expression of CAIX in HTB-35 cells under hypoxic conditions. The cells were incubated either in normoxia (21% O_2_ level) or in hypoxia (5% O_2_ level) with CA, Met or both compounds for 24 h. qPCR analysis was performed to assess the expression of HIF-1α under normoxic and hypoxic conditions (**A**). The RNA expression of *HIF1A* was determined with the 2^−ΔΔ*C*t^ method. The mRNA level of *HIF1A* was significantly increased following hypoxia ((**A**), *p* < 0.01 vs. control). On the contrary, Met alone and together with CA downregulated *HIF1A* ((**B**), *p* < 0.01 vs. control for Met, *p* < 0.01 vs. control for CA/Met). RT-PCR analysis revealed that in hypoxia the expression of CAIX significantly decreased in cells exposed to Met and CA/Met ((**C**), *HPRT1* was a reference gene). Data shown here are representative of three experiments performed with similar results (**C**).

Considering that transcription factor HIF-1α plays a primary role in mediating EMT program induced by hypoxia, we hypothesized that Met might influence the expression of HIF-1α protein. The qPCR analysis revealed that after 24 h of incubation of HTB-35 cells at 5% of O_2_ level, the expression of *HIF1A* significantly increased, when compared to cells kept under normoxic conditions (21% O_2_ of level), as presented in Figure 8A. Following exposition of cells to Met, the mRNA for *HIF1A* was significantly decreased (Figure 8B, *p* < 0.01 vs. control). What is more, as HIF-1α controls the expression of *CAIX*, in response to limited oxygen supply HIF-1α may cause the transcriptional activation of *CAIX* [15]. In conditions of chronic hypoxia, which develops in growing tumors, CAIX contribute to extracellular acidosis, which further promotes pro-metastatic cascade inducing cancer cell migration [16]. The results showed that following exposition of HTB-35 cells to Met in hypoxic conditions, mRNA for *CAIX* was downregulated, as measured by RT-PCR (Figure 8C).

## 3. Discussion

EMT has recently been regarded to play a crucial role in the mechanism underlying tumor spread. Therefore, EMT regulatory pathways represent potential new targets for inhibition of progression and metastasis of malignant cells [17]. Several studies have suggested that CA and its derivatives may restrain the spread of carcinoma cells via EMT inhibition [6,7]. Met has been previously demonstrated to be beneficial in gynecologic oncology [12,13] and to inhibit EMT in human cervical cancer cell lines [3]. Therefore, these compounds were used for current studies to investigate whether they may inhibit metastatic phenotype induced by TGF-β1 in C-4I and HTB-35 cervical cancer cells. Both cell lines expressed the epithelial characteristic, but the expression of epithelial and mesenchymal markers differed depending on a particular cell line. C-4I cells, which abundantly expressed E-cadherin and weakly Vimentin, were similar to normal cervical epithelium [18]. The exposition of cells to TGF-β1 caused upregulation of Vimentin and loss of E-cadherin. HTB-35 cells had high expression of vimentin and the stimulation of cells with TGF-β1 caused greater upregulation of Vimentin. At the same time, the amount of E-cadherin was minor in TGF-β1-treated and untreated HTB-35 cells. Vessey et al. reported HTB-35 cells to be E-cadherin negative [2] and Lee et al. suggested that some cervical carcinoma cells may concomitantly express both epithelial and mesenchymal markers [1,19]. Cheng et al. have shown that unstimulated HTB-35 cells may express E-cadherin [3]. The immunofluorescence analysis of E-cadherin level and its subcellular localization in HTB-35 cell, that we had previously performed, suggested that in wild-type HTB-35 cells E-cadherin might be weakly expressed, but the protein was withdrawn from intercellular junctions [20]. Our results showed that both compounds, CA and Met, had the potential to inhibit mesenchymal phenotype induced with TGF-β1 in cervical cancer cells, but each drug acted via various proteins in the particular cell line.

**Figure 9:**
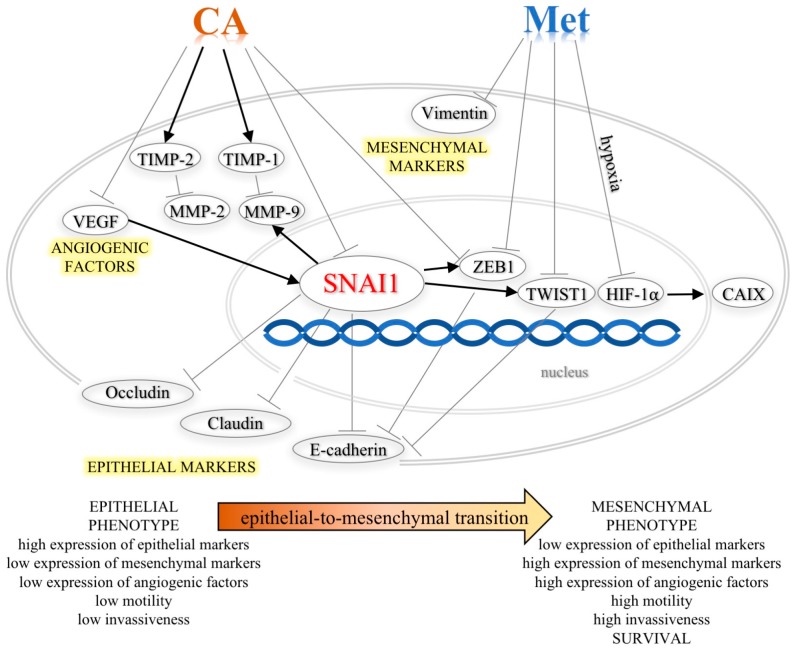
Schematic representation of the roles of CA and Met in inhibition of TGF-β1-induced EMT phenotype of cervical carcinoma cells. (↑—activation, Ⱶ—inhibition).

The signals from primary tumor associated stroma such as TGF-β1 may trigger changes in cytoskeleton reorganization and lead to the activation of nuclear transcription factors, ZEB1, TWIST1 and TWIST2, which, once activated, implement EMT program and promote invasiveness [21]. Many cell culture experiments and in vivo studies have demonstrated that inhibition of E-cadherin expression by its transcriptional suppressor SNAI1 is a key process driving EMT [22], also in cervical cancers [1]. It has been also reported that the inhibition of E-cadherin expression is positively correlated with the tumor stage and grade in cervical cancers in humans [23]. In the current study, the exposition of C-4I cells to CA at 100 µM caused downregulation of transcript and protein for SNAI1. We may speculate that this led to the increase in the expression of E-cadherin (Figure 9). The restoration of E-cadherin expression in cervical malignant cells results in decreased motility and invasiveness [24]. The functional test showed that CA treatment alleviated movement of C-4I cells and delayed wound healing. The transcription of tight junction molecules occludins and claudins may also be suppressed by SNAI1 [25]. Besides E-cadherin, these epithelial membrane proteins have been recognized to play essential role in cytokine-induced regulation of the tight junction and loss of their expression have been correlated with increased cancer cell movement and metastasis [26]. The results demonstrated the increased expression of Occludin and Claudin following CA treatment, which suggest that SNAI1 was no longer able to repress theses downstream proteins. In the current study the effect of CA on Snail expression was detected at the level of mRNA as well as the protein, thus we may speculate that CA can act directly on the transcription factor expression. However, we expect that the exact mechanism and possible upstream proteins involved in the process will be recognized in the following study.

The results demonstrated that CA downregulated the expression of transcription factor *ZEB1*, another repressor of E-cadherin. ZEB1 induces the promotion of EMT and in combination with other factors triggers metastasis [25] and is highly expressed in aggressive cancer cell lines [1] and the overexpression of the protein may be an indicator of poor prognosis in breast, pancreatic, and lung cancer [21]. What is more, a mutual regulation between SNAI1 and ZEB1 has been recently identified [25]; SNAI1, acting as a primary EMT regulator, may enhance expression of other transcription factors, including *ZEB1* and *TWIST* [27]. In metastatic cell nucleus, ZEB1 and TWIST1 proteins prevent E-cadherin gene transcription [1]. Interestingly, the action of CA on E-cadherin expression in C-4I cells was greater when co-treated with Met. At the same time, *TWIST1* was the molecular target regulated by Met in C-4I cells. Therefore, we may speculate that inhibition of these transcription factors under combined treatment may be a possible explanation for the enhanced transcription of junctional proteins. In line with these intracellular changes leading to lower expression of mesenchymal markers and higher expression of epithelial proteins, the incubation of C-4I cells with CA/Met inhibited TGF-β1-induced migration of the cells and caused the most visible scratch reduction.

Additionally, SNAI1 is a positive regulator of Matrix metalloproteinases (MMPs), which belong to a family of zinc-dependent extracellular matrix (ECM)–degrading proteases. Matrix metalloproteinase-2 (MMP-2) and metalloproteinase-9 (MMP-9) degrade structural proteins of invaded tissues and play a crucial role in metastasis of tumor cells and angiogenesis [28]. Although the expression pattern of MMPs varies depending upon the tumor origin, type and stage, numerous studies have shown that the suppression of MMPs synthesis results in the reduction in metastatic potential of cancer cells [29]. It was reported that overexpression of MMP-9 is associated with increased invasiveness of ovarian and breast tumors, which may lead to decreased survival in patients [30]. On the other hand, it was demonstrated that CA derivatives exert strong inhibitory effect on MMP-9 activity [31]. In the present study, CA suppressed *MMP-9* in C-4I cells in two independent ways. Firstly, the suppression of SNAI1 by the action of CA might result in reduced expression of *MMP-9*. Secondly, CA treatment of C-4I cells caused upregulation of a specific tissue inhibitor *TIMP-*1 controlling the degradative activity of MMP-9 [30]. It has been reported before that the increased synthesis of MMPs in tumor cells may be associated with enhanced angiogenic ability caused by overexpression of Vascular Endothelial Growth Factor A (VEGFA), which promotes metastatic potential of cancer cells [32]. In breast cancer cells, VEGF-A increases mRNA and protein level for SNAI1, resulting in repression of E-cadherin transcription [33]. Our findings suggest that CA, by suppressing *VEGFA*, may possibly contribute to the reduction of *SNAI1* transcription in cervical cancer cells, but further mechanistic study is needed.

While in C-4I line CA exerted the greatest effect in inhibiting the TGF-β1-induced metastatic phenotype; Met appeared to be an effective suppressor of EMT process in HTB-35 cells. We found that in HTB-35 cells Met at the concentration of 10 mM decreased the expression of mesenchymal marker Vimentin. As discussed above, Vimentin mRNA transcript and protein was higher in more aggressive HTB-35 cells than in C-4I cell line. This finding may explain in part the divergent effect exerted by compounds in the two cervical cancer cell lines. Moreover, Met exerted the greatest effect on motility of HTB-35 cells, as measured with functional scratch test.

In cervical cancers, motility of cells may be induced by hypoxia and enhanced acidosis of surroundings tissues [15,34]. The insufficiently oxygenated environment within solid tumors may induce Hypoxia-inducible factor 1 α (HIF-1α). The activation of HIF 1α results in upregulation of several proteins involved in migration and invasion that drives EMT program and helps neoplastic cells adapt to environmental stress [16]. In order to further elucidate the molecular action of Met in HTB-35 cells, we tested the effect of the compound in the hypoxic conditions that caused increased expression of transcription factor *HIF-1α*. The intracellular changes following activation of HIF 1α involve the induction its downstream protein Carbonic anhydrase IX (*CAIX*). CAIX can act as a survival factor that protects tumor cells against enhanced acidification of tissue microenvironment and through the ability to regulate pH of cancer milieu, it facilitates the migration of malignant cells. [35]. Due to its relevant role in metastasis, CAIX was also proposed as a potential therapeutic target in cervical cancers [15,36]. In our study, Met downregulated *HIF-1α*, which resulted in decreased transcription of *CAIX* (Figure 9). We may speculate that the inhibition of CAIX in HTB-35 cells exposed to Met can impair the invasive properties of cervical malignant cells.

Among a wide range of pharmacological and biochemical effects, CA [5,6,7,8,9,10,11] and Met [12,13] were shown to inhibit EMT in various cancers. Given that the net balance between EMT activators and suppressors determine further progress or EMT inhibition, we can speculate that downregulation of mesenchymal transcription factors and activation of epithelial molecules by the action of drugs may reverse mesenchymal phenotype of cervical carcinoma cells and impair their motility. Emerging data suggests that such a combinatory approach targeting different molecular mechanisms may be more effective in the cancer invasiveness reduction than standard one-drug therapy [37,38]. We have recently reported that CA may expand the anti-tumor effect of Met in human epithelial cervical carcinoma cells by regulation of metabolic reprogramming [39,40].

## 4. Materials and Methods

### 4.1. Cell Culture and Treatment

The human cervical cancer cell lines C-4I (ATCC designation CRL-1594) and HTB-35 (ATCC designation HTB-35, SiHa) were purchased from the American Type Cell Culture collection (LGC Standards-ATCC, Teddington, UK). C-4I cells were kept in Waymouth’s MB 752⁄1 medium (Life Technologies, Grand Island, NY, USA) and HTB-35 cells were grown in Dulbecco’s modified Eagle’s medium Lonza, Walkersville, MD, USA). Media were supplemented with 10% fetal bovine serum (BSA), (Eurex Sp z o.o., Gdansk, Poland) and 50 µg/mL of gentamicin (Sigma-Aldrich, Seelze, Germany). Cultures were grown at 37 °C in a humidified atmosphere of 5% CO_2_. For experiments, C-4I cells at a density of 2.5 × 10^5^ cells/mL and HTB-35 cells at a density of 1 × 10^5^ cells/mL were placed in cell culture plates (Sarstedt, Numbrecht, Germany) and incubated to reach adequate confluency. Following washing with PBS solution (Lonza), the medium in each well was replaced with a fresh medium with adequate volumes of stock solution of CA (100 µM, Sigma-Aldrich), Met (10 mM, Sigma-Aldrich) or both chemicals for 48 h. Each culture was prepared with or without addition of 10 ng/mL TGF-β1 (PeproTech, Rocky Hill, NJ, USA). For cell counting, the cells were cultured to full confluency and then exposed to compounds with or without TGF-β1 for 24, 48 and 72 h. Control cells were grown in medium with addition of solvents (untreated cells), or solvents and 10 ng/mL TGF-β1 (TGF-β1-treated cells). Each experiment was conducted in triplicate. The number of cells was assessed by automatic cell counter Countess (Gibco Laboratories, Grand Island, NY, USA).

### 4.2. Immunoblotting

After 24 h of incubation with compounds, the cells were harvested and extracts formed by the addition of ice-cold M-PER buffer (Thermo Fisher Scientific Inc., Waltham, MA, USA) with a protease inhibitor cocktail (Merck, Darmstadt, Germany). Total protein was measured by the Bradford method. 20 μg of protein was separated on 10% SDS-polyacrylamide gel and transferred to PVDF membrane for Western blotting. The membranes were blocked with buffer contained 1% BSA in TBST (20 mM/L of Tris-hydrochloride, pH 7.5, 150 mM/L NaCl, 0.05% Tween 20; BioRad, Laboratories, Hercules, CA, USA) as reported previously [39]. Then the membranes were incubated overnight with primary antibody in Tris-buffered saline (TBS) containing 0.1% Tween 20% and 1% BSA, followed by extensive rinses and a 1h incubation with HRP-linked secondary antibody (1:4000). The following primary antibodies were used for experiments: anti-E-cadherin (Cell Signaling, Danvers, MA, USA, dilution 1: 1000), anti-Vimentin (Cell Signaling, dilution 1: 1000), anti-SNAI1 Santa Cruz Biotech (Santa Cruz, CA, USA, dilution 1: 500. β-actin (1:1000, Cell Signaling) was the loading control. The secondary HRP-conjugated antibodies were purchased in Santa Cruz Biotch. The specific immunoreactivity of each protein was measured by enhanced chemiluminescence and developed using the Super Signal West Pico Chemiluminescent Substrate Kit, Pierce Chemical, Rockford, IL, USA) using Gel Logic Imaging System 1500 (Kodak; Molecular imaging System Corestea Health Inc., Rochester, NY, USA).

### 4.3. Quantitative Polymerase Chain Reaction (qPCR) 

The total RNA was extracted using Universal RNA purification Kit (EURx, Poland), according to vendor’s protocol. The reverse polymerase transcription of mRNA was performed using MMLV reverse transcriptase (Promega, Madison, WI, USA) according to the manufacturer’s protocol using ProFlex PCR System (Applied Biosystems, Foster City, CA, USA).

The real-time qPCR was performed in the QuantStudio 7 Flex (Applied Biosystems, Foster City, CA, USA) using Blank qPCR Master Mix (EURx) and the following Taq-Man human probes (Applied Biosystems): CDH1 (Hs01023894_m1), VIM (Hs00185584_m1), CTNNB1 (Hs0017025_m1), OCLN (Hs00170162_m1), CLDN1 (Hs00221623_m1), SNAI1 (Hs00195591_m1), ZEB1 (Hs00232783_m1), TWIST1 (Hs00361186_m1), TWIST2 (Hs02379973_s1), DES (Hs00157258_m1), MMP-2 (Hs00234422_m1), MMP-9 (Hs00234579_m1), TIMP-1 (Hs00171558_m1), TIMP-2 (Hs00234278_m1), VEGF (Hs00173626_m1), HIF1A (Hs00153153_m1) GAPDH (Hs99999905_m1). The data were normalized against GAPDH transcript as a reference gene and levels of RNA expression were determined with the 2^−ΔΔ*C*t^ method.

### 4.4. Wound Healing Migration

Alteration of cell migration induced by CA, Met and CA/Met in cultures of C-4I and HTB-35 cells was estimated by means of wound healing migration (alteration of two-dimensional cellular movement). The cells were cultured to sub-confluency in 12-well culture dishes and then a scratch was made on the monolayer of cells with a sterile 10 µL plastic pipette. After washing of floating cells with PBS, the medium in each well was replaced with a new one containing addition of adequate amounts of tested compounds with/without addition of TGF-β1. Cell movement into the wound area was photographed at the initiation of the experiment (0 h) and after 24 h under an inverted light microscope (Olympus IX-70 microscope with fluorescence, Olympus, Hamburg, Germany) from the exact same location as the first picture. The area of the scratch wound was measured using Image J (v1.44; National Institutes of Health, Bethesda, MD, USA) and the migration rate was quantified as a rate of the scratch reduction (scratch area at 0 h–scratch area at 24 h) and the data was calculated as the average of 3 fields as described therein [41].

### 4.5. Hypoxia Conditions

HTB-35 cells were seeded into 6-well plates (Sarstedt) at a density of 1 × 10^5^ cells/mL. After 24 h fresh medium with addition of CA, Met or both compounds were used and cells were incubated either in normoxia (21% O_2_ level) or hypoxia (5% O_2_ level) for 24 h. Afterwards, the cells were harvested for RNA isolation.

### 4.6. Reverse Transcription-Polymerase Chain Reaction (RT-PCR)

Total RNA was isolated from cells using the RNeasy Mini kit (Qiagen, Hilden, Germany) and the quantity of total RNA was measured using a NanoDrop ND-1000 Spectrophotometer (NanoDrop Technologies, Wilmington, DE, USA). cDNA synthesis was performed using oligo (dT) 15 primer and GoScript Transcriptase according to manufacturer’s instructions (Promega GmbH, Mannheim, Germany). The PCR mixture contained: 1.5 uL of cDNA, 1× PCR buffer (Sigma Aldrich) 2.5 mM Magnesium Chloride (Sigma Aldrich), 0.2 mM dNTPs (Sigma Aldrich), 0.2 uM of each primer (Sigma Aldrich), and 1.25 U JumpStart™ Taq DNA Polymerase (Sigma Aldrich). The cDNA was amplified by PCR using the MJ Research PTC-200 Thermal Cycler with the following primers: CAIX (Carbonic Anhydrase 9) forward (5′-TACAGCTGAACTTCCGAGCG-3′), CAIX reverse (5′-CTAGGCTCCAGTCTCGGCTA-3′), HPRT1 (Hypoxanthine-Guanine Phosphoribosyltransferase) forward (5′-TGGCGTCGTGATTAGTGATG-3′), HPRT1 reverse (5′-TATCCAACACTTCGTGGGGT-3′). The PCR conditions for all analyzed genes were as following: denaturing at 95 °C for 5 min, followed by 30 cycles of 30 s at 95 °C, 30 s at 58 °C and 30 s at 72 °C. The reaction was completed for 10 min at 72 °C. The PCR reaction was evaluated by checking the PCR products on 1.5% *w*/*v* agarose gels. Bands were normalized by use of HPRT1 to correct for differences in loading of the cDNAs.

### 4.7. Statistical Analysis

The experimental data were shown as mean ±SD. Analysis was performed using one-way ANOVA followed by Duncan post-hoc test. *p* values < 0.05 and *p* < 0.01 were considered statistically significant. Calculations were carried out using the commercially available packages Statistica PL v.10 (StatSoft, Tulsa, OK, USA).

## 5. Conclusions

In conclusion, treatment of human squamous cancer cells with CA and with Met suppressed the motility of cells and the effect depended on a particular cell line. Both compounds regulated EMT process in C4-I and HTB-35 cells by interfering with different molecular targets. In TGF-β1-stimulated C4-I cells, CA suppressed the expression of mesenchymal transcription factor SNAI1 which resulted in enhanced expression of epithelial markers E-cadherin, Occludin and Claudin. Additionally, CA blocked *MMP-9* and upregulated *TIMP-1*, a specific inhibitor of MMP-9. In HTB-35 cells stimulated with TGF-β1, Met decreased the expression of Vimentin. By suppressing hypoxia master regulator *HIF-1*α, Met caused downregulation of *CAIX*, an enzyme involved in metastasis of aggressive malignant cells. In this study we showed that CA and Met inhibited EMT process in cancer cells via different mechanisms. However, when applied together, compounds exerted a greater effect on EMT than each compound alone.

This is the first report revealing that CA alone and CA co-treated with Met may reverse the mesenchymal phenotype of TGF-β1-treated cervical tumor cells and we believe that the use of the two small molecules may be considered a potential therapeutic approach for metastatic cervical cancer.

## Supplementary Materials

Supplementary materials can be found at www.mdpi.com/1422-0067/19/1/266/s1.
