# Supplementary material for: Caffeic Acid and Metformin Inhibit Invasive Phenotype Induced by TGF-β1 in C-4I and HTB-35/SiHa Human Cervical Squamous Carcinoma Cells by Acting on Different Molecular Targets
Source: Int J Mol Sci. 2018 Jan 16;19(1):266. doi: 10.3390/ijms19010266 (PMC5796212; doi:10.3390/ijms19010266)
Supplement: Supplementary file 1 [file ijms-19-00266-s001.zip › Supplement 2.docx]

**Supplement 2**

**Figure S2.** The effect of Caffeic Acid (CA) and Metformin (Met) on the expression of EMT regulatory proteins in human cervical squamous cell cancer lines C4-I and HTB-35 after 24 h of exposure to compounds. The cells were incubated with or without TGF-β1 (10 ng/mL) for 24 h. CA was applied in concentration of 100 μM and Met in concentration of 10 mM. Analysis of mRNA level for epithelial marker E-cadherin (*CDH1*) was shown in panel A and transcript levels for mesenchymal transcription factor *SNAI1* was presented in panel B (in C4-I cells). In panel C, the expression of mesenchymal marker *VIM1* in HTB-35cells was shown. The data were normalized against GAPDH transcript as a reference gene and levels of RNA expression were determined with the 2-^ΔΔCt^ method (**p*<0,05 and ***p*<0,01 vs control, ^#^*p*<0,05 and ^##^*p*<0,01 vs control with TGF-β1). Experiments were repeated three times with similar results and presented as mean values ± SD.
