# Supplementary material for: Caffeic Acid and Metformin Inhibit Invasive Phenotype Induced by TGF-β1 in C-4I and HTB-35/SiHa Human Cervical Squamous Carcinoma Cells by Acting on Different Molecular Targets
Source: Int J Mol Sci. 2018 Jan 16;19(1):266. doi: 10.3390/ijms19010266 (PMC5796212; doi:10.3390/ijms19010266)
Supplement: Supplementary file 1 [file ijms-19-00266-s001.zip › Supplement 1.docx]

**Supplement 1**

**Effect of CA and Met on C4-I and HTB-35 cervical cancer cells growth**

The cells were grown to full confluency and then treated with CA at 100 µM and/or Met at 10 mM for 24, 48 or 72 h. In parallel, the cultures with addition of tested compounds and TGF-β1 were prepared. None of the tested compounds affected C4-I or HTB-35 cells growth after 24 and 48 h. CA at 100 µM caused significant reduction of C4-I cells growth with and without presence of TGF-β1 (*p*<0.05 vs. control for TGF-β1-unstimulated cells, *p*<0.05 vs. control for TGF-β1-stimulated cells) and had no effect in HTB-35 cell line after 72 h. Met alone and when applied with CA decreased growth of HTB-35 cells after 72 h (*p*<0.05 vs. control and *p*<0.01 vs. control, respectively). The combined treatment significantly inhibited growth of C4-I cells after 72 h (*p*<0.05 for CA/Met vs. CA, *p*<0.05 for CA/Met vs. Met).

**Figure S1.** For cell counting the cells were exposed to compounds with or without TGF-β1 (10 ng/mL) for 24, 48 and 72 h. Caffeic Acid (CA) was applied in concentration of 100 μM and Metformin (Met) in concentration of 10 mM. Each culture was incubated with or without addition of 10 ng/mL TGF-β1. The number of cells was assessed by automatic cell counter Countess (Gibco Laboratories, NY, USA).
