# Supplementary figures and images for: Caffeic Acid and Metformin Inhibit Invasive Phenotype Induced by TGF-β1 in C-4I and HTB-35/SiHa Human Cervical Squamous Carcinoma Cells by Acting on Different Molecular Targets
Source: Int J Mol Sci. 2018 Jan 16;19(1):266. doi: 10.3390/ijms19010266 (PMC5796212; doi:10.3390/ijms19010266)

**- TGF- $\beta$ 1****+ TGF- $\beta$ 1****A**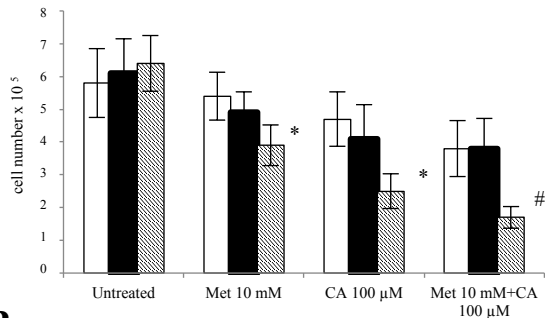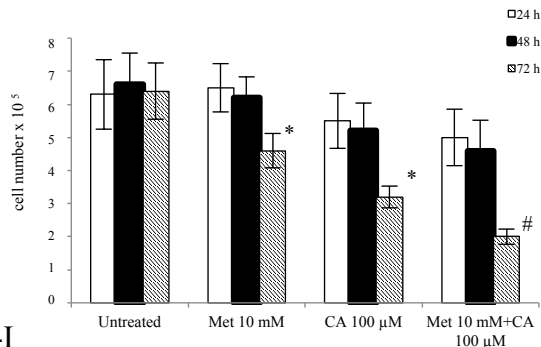**C4-I****B**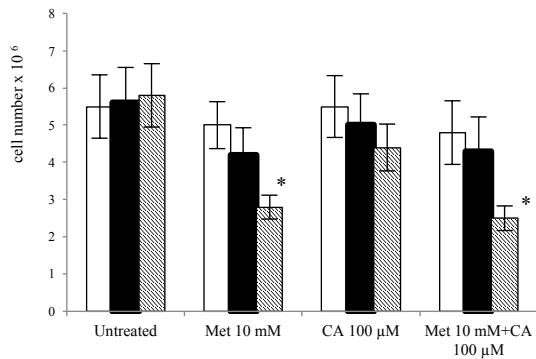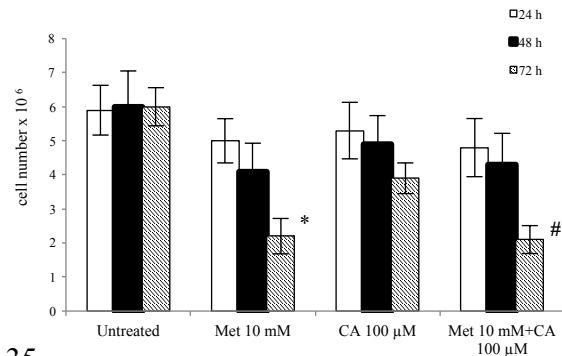**HTB-35**

Supplement: Supplementary file 1 [file ijms-19-00266-s001.zip › Fig S1.pdf]

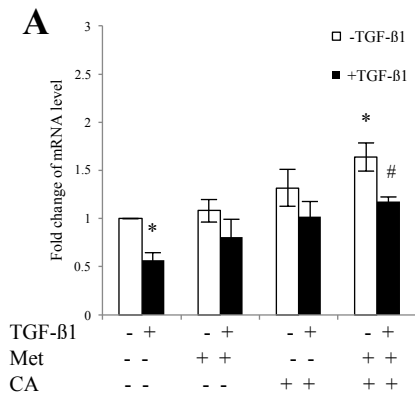

*CDH1*

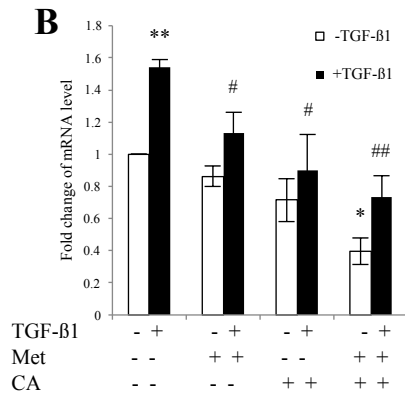

*SNAIL*

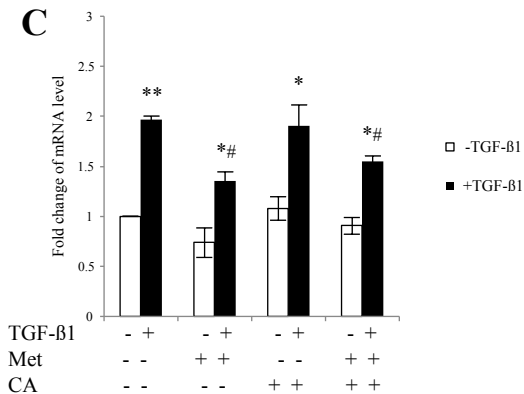

*VIM1*

Supplement: Supplementary file 1 [file ijms-19-00266-s001.zip › Fig S2.pdf]
